# A Fault-Tolerant Transmission Scheme in SDN-Based Industrial IoT (IIoT) over Fiber-Wireless Networks

**DOI:** 10.3390/e24020157

**Published:** 2022-01-20

**Authors:** Qinbin Zhou, Taotao Zhao, Xiaomin Chen, Yuesheng Zhong, Heng Luo

**Affiliations:** 1 School of Information Science and Technology, Nantong University, Nantong 226019, China; 2010310021@stmail.ntu.edu.cn (Q.Z.); fatt1791970098@gmail.com (T.Z.); 2 BMW Group, 80788 Munich, Germany; yuesheng.zhong@bmw.de; 3 Jiangsu Province Key Laboratory of Intelligent Building Energy Effciency, Suzhou University of Science and Technology, Suzhou 215009, China; hluo@mail.usts.edu.cn

**Keywords:** network coding, fiber-wireless network, industrial Internet of Things, software-defined network, fault-tolerance, parallel transmission

## Abstract

Driven by the emerging mission-critical and data-intensive applications in industrial intelligent manufacturing, the software-defined network (SDN) based fiber-wireless access network (FiWi) is attracting considerable attention thanks to its capability of central control and large bandwidth. However, the heterogeneity of the network leads to new challenges, since the packet loss can be caused either by the poor channel quality of wireless links or network component failures. A novel and adaptive mechanism combining sparse random linear network coding with parallel transmission (SNC-PT) is proposed to achieve the fault-tolerance against high packet loss rate and any network element malfunction. We illustrate the benefits of using the SNC-PT mechanism to improve fault tolerance by characterizing the network performance with respect to the completion time and goodput along with its relationship to channel quality and node failures. We show that significant performance gains can be obtained in comparison with conventional uncoded transmission based on transmission control protocol (TCP). The simulation results show that the SNC-PT mechanism is fault-tolerant, while it can significantly shorten the data transmission completion time to at least 12% of the baseline and increase the goodput by about 10% compared to other coding schemes such as random linear network coding.

## 1. Introduction

The Internet of Things (IoT) has become popular in the industrial sector to support rapidly developing smart factories, where heterogeneous sensors, intelligent systems, machines, etc., are connected. Communication technologies, such as fifth generation (5G) technologies, IPv6 over low-power wireless personal networks, and passive optical networks, have also paved the way for gradually adopting the IoT in industrial settings, which is now referred to as the industrial IoT (IIoT). Smart manufacturing lines and systems increasingly exchange huge amounts of data generated by intelligent terminals, machines, sensors, control units, users, and so on. The latest trends in industrial applications are progressively aggravating communication traffic in such scenarios. Therefore, a high-capacity, low-latency, reliable, and flexible access network is essential for IIoT. However, the heterogeneity of the applications and services in the IIoT makes it difficult for legacy access networks with the one-size-fits-all design concepts to satisfy the requirements [1]. The hybrid fiber-wireless (FiWi) access network, also known as wireless-optical broadband access network (WOBAN), was proposed to integrate the complementary features of optical fiber and wireless access technology [2,3].

The FiWi network encompasses a composite of passive optical network (PON) technologies and wireless technologies. It can provide end-user devices with better quality of service (QoS), more flexible access, higher bandwidth capacity and a better quality of experience (QoE) compared to traditional access technologies. However, currently, SDN technology is playing a significant role in the IIoT. It can define and control networks through software programming to make networks more flexible [4], which makes it possible to satisfy the growing requirements of the IIoT. However, there remain several challenging areas requiring concentrated research efforts before standardization can take place [5]. Fault-tolerance is one of the important topics. On the one hand, due to dynamic noise, interference, and dynamic channel effects, handling packet loss is a fundamental challenge in wireless networks; on the other hand, any component in the FiWi network may break down, resulting in communication interruptions.

To date, research efforts have been focused either on the wireless sector or PON. Few methods have been reported which can cover both sectors at the same time while considering the characteristics of industrial IoT scenarios. In wired networks, rerouting or presetting a backup path are common solutions [6,7,8,9]. Rerouting the interrupted traffic by finding a backup path with sufficient resources requires a long recovery time, which is not suitable for time-sensitive applications in the IIoT. Solutions that preset backup fibers and nodes are challenged by low network utilizations, extra deployment costs, etc. To address these issues, researchers have proposed to deploy a certain number of sleeping wireless optical network units (WONUs) in the network. The sleeping nodes are only woken when some components break down. The interrupted traffic is resumed by using the backup nodes [8,9]. Although these methods can achieve survivability and reliability to some extent, their recovery time and resource utilization cannot meet the growing requirements of the IIoT. It is very important to study new fault-recovery schemes to improve the overall performance of the network. In this paper, we propose a novel fault-tolerant transmission scheme based on network coding and parallel transmission. It is designed for an SDN-based IIoT over a FiWi network, which can tolerate node failures as well as packet losses on wireless links without retransmissions and long recovering time.

**Figure 1:**
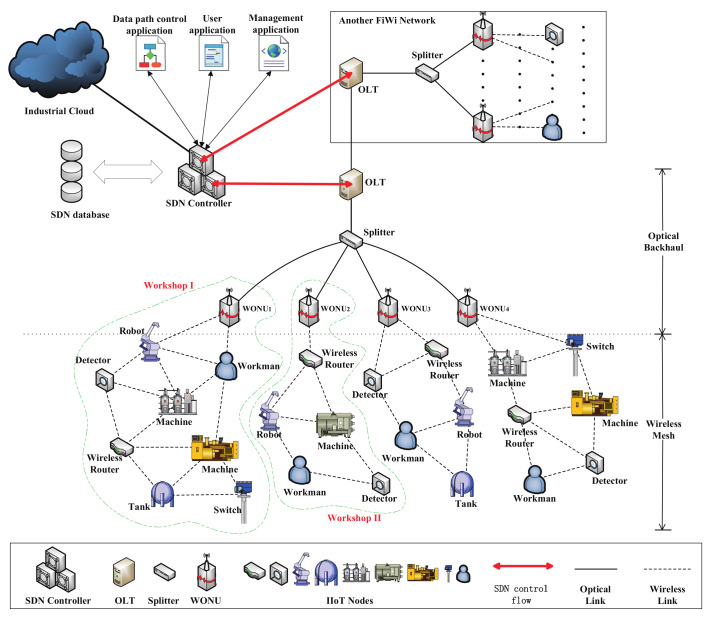
Reference architecture.

To simplify the illustration of the proposed scheme, the reference architecture is presented in Figure 1. The network can be divided into two parts, referred to as front end and back end, respectively. The industrial wireless mesh network (WMN) part is at the front end. It is composed of intelligent end systems, machines, wireless routers, robots, etc. The optical network part is located in the back end. Unlike standard PON networks where the optical network unit (ONU) provides services for users directly, in the FiWi network, the ONU is connected with the base station of the wireless front end and equipped with a wireless transceiver function; these ONUs are called wireless ONUs (WONUs) and serve as the gateway connecting the PON part and the WMN part [10]. The wireless front-end adopts a cluster structure, which aligns with the actual situation in factories. Moreover, a cluster network has more advantages in scalability, flexibility, and management [11]. In each cluster, a WONU, as the cluster head (CH), is connected with many IIoT devices that form a small wireless mesh network in a certain area. In thinking towards networks in industrial settings, all the intelligent nodes are typically connected to a single optical line terminal (OLT) via multiple WONUs. An SDN controller makes it feasible to define and enforce consistent policies across both wired and wireless connections, resulting in great potential for seamless and fully integrated FiWi networks and industrial IoT networks.

The proposed scheme is tailored for such settings mentioned above and is designed to mitigate against any network element malfunctions, excluding OLT failures. To enhance the fault tolerance, the SDN controller makes decisions on the packet encoding process and path selection process according to the delay of the links, the reliability requirements, and the accessible minimum cost of recovery. The following contributions are made in this paper:

- An effective parallel transmission mechanism is proposed, combined with sparse network coding (SNC-PT) in an SDN-based IIoT over a FiWi network. With network coding, the destination node will be able to recover the original packets as long as it receives enough encoded packets.
- We systematically analyze the minimum number of redundant encoded packets and devise two network coding methods combined with parallel transmission. Various scenarios are studied, and solutions are proposed accordingly.
- The performance of the proposed mechanism is compared with conventional transmissions using a transmission control protocol (TCP) with retransmissions. Simulation results show that our mechanism has lossless fault tolerance and recovers interrupted traffic rapidly due to the combination of network coding and SDN technology in the FiWi network.

The rest of this paper is organized as follows. Section 3 introduces the system model and analyzes the proposed mechanism, including routing and coding strategies. Section 4 focuses on performance evaluations. Finally, Section 5 concludes the paper.

## 2. Related Works

The development of the IIoT enhances the advantages of advanced manufacturing machinery and intelligent control, leading to a more intelligent and interconnected industry [12]. Along with the new elements and advantages, different challenges and requirements are also brought up by the IIoT [13]. Among which, fault tolerance, including survivability and reliability, plays an important role in the field of industrial networks [14].

A virtualized access network was designed in [15] to provide robust IoT services even when the network components fail. An access network design combining FiWi and edge computing nodes is proposed in [16], and the experimental results show that it can provide large-scale regional and low-latency cloud services for IoT applications. Because most industrial IoT systems are deployed in complicated environments, many centralized scheduling methods [17,18] and rhythmic task models [19] have been proposed to address various internal and external disturbances. Unfortunately, these approaches are almost all based on a centralized architecture to meet the requirements of end-to-end latency, resulting in limited scalability [20]. SDN, as a novel enabling technology, is gaining increasing attention due to its capability to overcome the shortcomings of traditional network infrastructures. It clearly separates the control plane from the data plane so that all the decision-making procedures are performed by SDN controllers instead of routers or switches in traditional networks, leading to efficient communications [21]. Relevant literature is reviewed in detail in [22], identifying the main requirements of today’s industrial networks and solutions about security, control, and management through SDNs. The solutions to use SDN in industrial IoT scenarios are usually very specific. For example, SDN solves the problems of adaptive transmission, fault tolerance, or security through utilizing new protocols [23], models calculating redundant paths [24], and anomaly detection systems [25]. In [26], an IIoT-based SDN platform was proposed to address the strong demand for seamless data transmission in smart grid systems, which are also known as software-defined industrial Internet of Things (SDIIoT) [27,28]. This architecture has been proven to reduce the complexity of implementation, provide dynamic reconfiguration, and improve robustness. These above solutions are all targeting specific problems.

Other researchers are committed to studying a comprehensive solution by improving traditional networks with SDN in IIoT scenarios [29]. By combining a group of SDN applications focusing on different specific aspects of IIoT communication operation, the researchers in [22] proposed a comprehensive SDN solution called the SDN ecosystem proposal, which is able to meet the requirements of reliability, adaptability, fault tolerance, and being in real-time in modern industry. Although this type of solution is a significant improvement of the current IIoT network, it is difficult to implement. In particular, emerging data-intensive and delay-sensitive applications in the industrial area require a fault-tolerant transmission network. The FiWi network can provide large bandwidths and flexible access, which is regarded as a potential candidate. However, the heterogeneity of the network makes fault-tolerant transmission more challenging.

To this end, research efforts in fault-tolerant transmission have been focused on enhancing survivability against component failures, or improving reliability in the case of lossy links. In terms of improving the reliability of lossy links, network coding has been proposed as an effective solution; communications can approach the max-flow capacity of the network and improve the throughput [30,31,32]. Studies have been carried out on implementing random linear network coding (RLNC) in SDN-based networks [33,34,35]. Simple intraflow network coding methods can produce considerable gains in the throughput and delay in PONs [36]. Some researchers in [37] try to combine random/sparse linear network coding and retransmission techniques to improve the reliability of communication. Although the fault tolerance of the network was guaranteed due to the network coding and retransmission, the performance of end-to-end latency was not sufficient to meet the requirements of IIoT. In this paper, we introduce parallel transmission technology to address this issue.

Solutions that prevent failures can be categorized into two groups. The first solution is to reroute the interrupted traffic through the rest of the network, which may recover the data transmission successfully within an acceptable recovery time. A dynamic network formation (DNF) was proposed in [6] to improve survivability by designing routing algorithms to avoid areas with a high possibility of failure or congestion. A joint use of an SDN controller in the FiWi-Edge IoT mixed space was proposed in [7] to reroute the traffic in the Edge-IoT domain to reduce the latency and improve the survivability. In [38], the authors proposed an optimization method to create a survivable FiWi access network with an integrated small cell and WiFi, which provides connectivity and protects the network by rerouting traffic over WiFi. The second solution category contains mainly protection methods based on preset backup resources, which are known to have issues of high cost. To address this issue, solutions were proposed to use “wake-up” strategies. A certain number of sleeping ONUs are deployed in the network and waken up only when some components break down [8,9]. However, because these backup ONUs do not work during normal communications, the resource utilization is still relatively low. In order to reduce the redundant backup resources, a resource selection and deployment optimization mechanism was proposed in [39] using a simulated annealing algorithm and greedy algorithm to reduce the costs and improve the survivability. The framework proposed in [40] can reroute the traffic through the wireless paths from the interrupted ONU to the backup ONUs when a certain distribution fiber fails to support a survivable FiWi network.

## 3. The Principle of the SNC-PT Mechanism

In general, there are three levels of failures in FiWi networks [41]:

- OLT-level failures: the failures of OLT, feeding fiber, and optical splitters;
- ONU-level failures: the failures of the WONUs and distribution fibers;
- End-system failures: the failures of the IIoT systems, wireless routers.

Because OLT-level failures cannot be handled in the same FiWi network, they are not considered in this paper. In practical industrial scenarios, a typical manufacturing setting, e.g., a workshop, is deployed as a cluster, where the WONU is the cluster header. There is an IIoT access gateway, i.e., a WONU enables the access of various industrial equipment and sensors. The SDN controller allows the control and transmission of communication data and protocols of different devices. OLT converges and transmits industrial data to the application layer of the cloud platform. In this paper, we propose a novel parallel data transmission mechanism called SNC-PT, combined with sparse network coding and parallel routing in an IIoT network to achieve fault tolerance. This is a design to prevent poor channel quality and any network element malfunctions, excluding OLT failures.

### 3.1. Parallel Routing and Coding Strategy

Since OLT-level failures are not considered in this paper, the path delay is counted from the WONU to the end-nodes. The delays of downstreaming and upstreaming transmission are similar. In the following analysis, only downstreaming will be discussed.

#### 3.1.1. Delay Analysis

An end-to-end delay from the WONUs to an IIoT node consists of four parts: the propagation delay, transmission delay, slot-synchronization delay, and queuing delay. A transmission delay and slot synchronization delay depend on the capacity of wireless links and the average packet size. A queuing delay is related to packet arrivals and service rates on wireless links. Assuming that the average size of the packet is $\frac{1}{\mu }$, the capacity on the *i*th wireless link is denoted as $C_{i}$, and $\lambda_{i}$ is the packet arrival rate on the *i*th wireless link. *E* is the set of wireless links so that i∈[1,|E|]. The delay on the *i*th wireless link is shown in (1) [33]:

(1) $$\begin{array}{cc} d_{i} & =\frac{1}{\mu C_{i}}+\frac{1}{2\mu C_{i}}+\frac{\lambda_{i}}{\mu C_{i}\left(\mu C_{i}-\lambda_{i}\right)}\\ \; & =\frac{1}{2\mu C_{i}}+\frac{1}{\mu C_{i}-\lambda_{i}} \end{array}$$

Assume that $W_{j}$ is the set of wireless links that belong to the *j*th parallel routing path, where j=1,2,3,.... Therefore, the total wireless delay on the *j*th routing path is described in (2):

(2) $$D_{Wpart}^{DOWN}=\sum_{i=1}^{\left|E\right|}d_{i},\;\mathrm{if}\;\mathrm{link}\;i\;\in W_{j}$$

The link delay is set as a weight, which will be used in the path selection. The SDN controller selects parallel routing paths in the network as required for the encoded packets that need to be transmitted to the same destination node.

#### 3.1.2. Network Coding Process

Assume that $S=\{S_{1},S_{2},...,S_{m}\}$ is the set of *m* original packets and $S_{i}$ stands for the *i*th packet. Similarly, the set $D=\{D_{1},D_{2},...,D_{M}\}$ represents the *M* encoded packets, and the *j*th encoded packet is noted as $D_{j}$. In the encoding process, *m* original packets are coded into *M* encoded packets according to the principle of random linear network coding, as shown in (3), where $g_{j,i}$ is the coding coefficient selected uniformly and randomly from the Galois field, and $\left[g_{j,1},g_{j,2},...,g_{j,m}\right]$ is the encoding vector (EV) and will be transmitted with encoded packets in the form of a packet header. The encoding process is shown in (3):

(3) $$D_{j}=\sum_{i=1}^{m}g_{j,i}S_{i}$$

The intermediate nodes on the path only forward the encoded packets. Since each encoded packet is a linear combination of *m* original packets and the coding coefficients are carried in the header, the target node can decode the packets by using a Gaussian elimination method to solve the linear equations. As long as the target node receives at least *m* linearly independent encoded packets from the *N* parallel routing paths, it is able to finish the decoding process successfully and recover the *m* original packets. According to the optimal packet allocation method, the number of packets transferred on each routing path is equal to $\frac{M}{N}$, where *M* is the number of encoded packets.

In fact, a larger *M* can increase the tolerance of the packet loss and make decoding easier under the same conditions. However, it will increase the network load. A trade-off between fault tolerance and network efficiency is important. The number of redundant packets depends on the number of failures in the system. So, we take a transmission scenario with two random failures as an example. To ensure fault tolerance, the number of redundant packets is calculated as follows:

Assume that the *N* parallel routing paths are noted as set $p=\{p_{1},p_{2},...,p_{N}\}$ and the links belonging to the *i*th routing path are noted as set $p_{i}=\left\{e_{1},e_{2},...\right\}$. To ensure that the FiWi can recover rapidly and losslessly when any two of the *N* routing paths break down with IIoT end-system failures, the total number of packets transferred on any N−2 routing paths is supposed to be no less than *m*. In other words, the destination IIoT node will be able to obtain enough encoded packets as long as the number of packets transferred on each routing path is at least $\frac{m}{N-2}$, which can be calculated by (4):

(4) $$E_{P}\times \left(N-2\right)\geq m,$$

where $E_{P}$ is the number of encoded packets transferred on each path, also known as $\frac{M}{N}$, according to the optimal packet allocation. Therefore, the total number of encoded packets is limited by (5):

(5) $$M\geq \frac{N}{N-2}m$$

However, in real situations, packet loss exists on wireless links. If each routing path only passes the minimum number of encoded packets as analyzed above, the destination node will very likely not be able to receive enough packets for decoding. Therefore, the total number of encoded packets should be increased to compensate for the packet loss caused by the instability of the wireless links. Assume that *R* is the number of redundant encoded packets, which is related to the packet loss rate of each wireless link. Therefore, the number of encoded packets transferred on each routing path can be noted as $\frac{M+R}{N}$.

Assume that $L_{j}$ stands for the packet loss rate of the *j*th wireless link in the *i*th parallel routing path $p_{i}=\left\{e_{1},e_{2},...,e_{j},...\right\}$. The probability for a packet to be completely transferred from OLT to the IIoT device through the *i*th routing path $p_{i}$ can be noted as $\prod_{e_{j}\in p_{i}}\left(1-L_{j}\right)$. Thus, the expected number of encoded packets noted as $n^{i}$ transferred on the *i*th routing path is limited by inequality (6):

(6) $$n^{i}\times \prod_{e_{j}\in p_{i}}\left(1-L_{j}\right)\geq \frac{m}{N-2}$$

According to the above inequality, considering the packet loss rate on wireless links, the minimum number of encoded packets can be derived as in (7):

(7) $$n_{min}^{i}=\left\lceil \frac{m}{\left(N-2\right)\times \prod_{e_{j}\in p_{i}}\left(1-L_{j}\right)}\right\rceil$$

Then, the minimum number of encoded packets transferred from the OLT, which can be noted as $M^{\prime }$, is calculated in (8):

(8) $$M^{\prime }=\sum_{i=1}^{N}n_{min}^{i}=\sum_{i=1}^{N}\left\lceil \frac{m}{\left(N-2\right)\times \prod_{e_{j}\in p_{i}}\left(1-L_{j}\right)}\right\rceil$$

Thus, the minimum number of redundant encoded packets *R* is shown in (9):

(9) $$R=M^{\prime }-M=\sum_{i=1}^{N}\left\lceil \frac{m}{\left(N-2\right)\times \prod_{e_{j}\in p_{i}}\left(1-L_{j}\right)}\right\rceil -\frac{N}{N-2}m$$

### 3.2. Fault-Tolerant Transmission Mechanisms

In the industrial Internet, most of the existing applications rely on the transmission control protocol (TCP) for reliable packet transport. When packet loss is caused by node/link failures or poor quality wireless links, it resorts to the retransmission of the missing packets. User datagram protocol (UDP) is known to be simpler and faster in comparison with TCP, whereas it has no guarantee of recovery and error-checking.

The proposed fault-tolerant transmission scheme takes advantage of UDP while using RLNC to ensure reliable transmission. Parallel routing and coding strategies are integrated into the SDN controller. Control messages including the routing information, coding redundancy factor, batch size, and batch transmission size (BTS), which will be introduced later, are directly sent to the specific devices by the SDN controller. In the downstream transmission, the OLT implements the NC process according to the instructions of the SDN controller and sends the encoded packets to the target node through parallel routing paths. The same goes for the upstream transmission.

In this section, we will introduce two fault-tolerant transmission mechanisms based on parallel routing and network coding. The UDP protocol is used in the packet transport. We first present the mechanism utilizing original random linear network coding. To overcome the issue of additional overhead and complexity caused by coding, we also propose a fault-tolerant transmission mechanism with sparse network coding. Both mechanisms are studied and analyzed.

#### 3.2.1. Fault-Tolerant Transmission with RLNC and Parallel Transmission (RLNC-PT)

Assume an *L*-hop lossy wireless path for transmission, in which the source node, denoted as *S*, has an *F*-bit message that needs to be sent to the destination node, denoted as *D*, through L−1 intermediate nodes. The *F*-bit message will be split into *m* original packets, which are $S=\{S_{0},S_{1},...,S_{m-1}\}$, and $S_{i-1}$ stands for the *i*th packet. Each original packet has $K=\frac{F}{m}$ bits. If *F* is not an integral multiple of *m*, zeros can be padded. At the *j*-th transmission opportunity, *S* will generate an encoded packet as in (10):

(10) $$R_{j}=\sum_{i=0}^{m-1}g_{j,i}S_{i},$$

where j=0,1,... and $g_{j,i}$ are the coding coefficients uniformly randomly selected from a finite field $F_{q}$ of size *q*. Each encoded packet has *K* bits. The encoding vector, which is denoted as $\left[g_{j,0},g_{j,1},...,g_{j,m-1}\right]$, is attached to the encoded packet in the form of a $mlog_{2}q$ bit header. Therefore, each transmission from the *S* sends a packet containing ($mlog_{2}q+K$) bits.

In the case of a UDP transmission, each encoded packet with its header generated by *S* is packaged with the corresponding headers and then passed through the routing paths with a probability of $\prod_{i=1}^{L-1}\left(1-l_{i}^{w}\right)$, where $l_{i}^{w}$ is the packet loss rate of the *i*-th wireless link on the transmission path. For each UDP message that is successfully received, destination node *D* obtains an encoded packet and related EV after removing the headers and then caches it. The cache space is set to no less than *m* encoded packets. Because UDP is a transport protocol with a message boundary, the packet received will always contain a complete encoded packet and EV.

The intermediate nodes on the routing path only forward the encoded packets. Thanks to the fountain feature of RLNC, the intermediate nodes and destination node do not need to acknowledge the received packets. Since each encoded packet is a linear combination of *m* original packets and the coding coefficients are carried in the header, the target node *D* can decode the packets by using the Gaussian elimination method by solving linear equations to perform the decoding process. As long as the target node receives no less than *m* linearly independent encoded packets, it will be able to decode and successfully recover the original packets.

#### 3.2.2. Fault-Tolerant Transmission with SNC and Parallel Transmission (SNC-PT)

Network coding causes additional complexity and overhead. In the case of RLNC, the coding overhead is ($mlog_{2}q+K)$ bits. To address this issue, fault transmission with a sparse network coding (SNC) is introduced, which is based on fixed size subsets. Coded packets are always a random linear combination of *d* source packets, where d<m. At the source node *S*, *d* original packets are selected from *m* original packets evenly and randomly. These packets are called a *batch*, and *d* is called the *batch size*. In the next *b* transmissions, the encoded packet sent by *S* is a random linear combination of the original packets in the *batch*, and *b* is called the *batch transmission size* (BTS). After *b* transmission, *S* reconstructs a random *batch* and cycles accordingly.

A synchronous pseudorandom number generator is deployed in each node to select packets, and they are grouped into a random *batch*. The coded packet carries the identifiers of the *batch* and *d* coding coefficients. The former enables the decoder to know the label of the original packets contained in the *batch* according to the pseudorandom number generator, and the latter is used to construct the corresponding linear equations for GE decoding. Compared with the RLNC, the *m*-dimensional EV corresponding to the coded packet contains at most *d* nonzero coding coefficients, so it is *s*parse in comparison with the conventional RLNC. The overhead of the SNC is ($dlog_{2}q+K)$ bits.

To alleviate the coupon collector problem [42], a precoder is added, inspired by the Raptor code [43]. Before the SNC process, the *m* original packets are precoded to m+c intermediate packets by using a systematic code. The set of intermediate packets can be denoted as $\left\{S_{0},S_{1},...,S_{m-1},C_{0},...,C_{c-1}\right\}$, where $C_{i}=\sum_{j=0}^{m}\omega_{i,j}S_{j}$ and i=0,1,...,c. $C_{i}$ are parity-check packets, $\omega_{i,j}$ are precoding coefficients selected from $F_{q}$ or its subfield, and *c* is the number of parity-check equations.

## 4. Performance Evaluation

In this section, we evaluate the proposed fault-tolerant transmission mechanisms in the ns-3 simulator. We create a network of 21×4 IIoT nodes and 4 WONUs on the edge and divide it into 4 clusters. Each cluster has 1 WONU as the cluster head and 21 IIoT nodes as the cluster members. The data flow follows a Poisson distribution. The bandwidth of the optical channel is set to 1 Gbps. The distance between the OLT and WONU is 1 km. The propagation delay on the optical links is 5 μs. The average rate of the wireless channel is 2 Mbps. In the simulation, OLT sends *F* = 1,344,000 bits, i.e., 168,000 bytes, of data to the target IIoT node, which is divided into m=160 source packets. Each packet contains 1050 bytes (K=8040 bits).

In the study, the performance of the proposed mechanisms in the scenario of no failures is used as the baseline. The proposed mechanisms with RLNC and SNC are studied in a scenario with the single failure. The performance of a conventional transmission based on TCP is also studied in the same scenario. Rerouting is adopted to ensure successful transmission between the source and destination. The performance metrics used in the simulation are listed as follows:

- *Completion Time*: This is the time of a complete transmission, which starts from the source node sending packets and ends when the destination node receives all the packets successfully. In case of node failures or poor channel quality, recovery of the lost packets leads to a long completion time. The longer the completion time, the poorer the performance of the transmission.
- *Average Throughput*: This metric is the average rate of data successfully delivered over the communication channel.
- *Goodput*: This metric reflects the effective amount of communication data successfully received at the destination node in unit time.
- *Average Delay*: This metric represents the average time required for each packet to be successfully received by the destination node.

In wireless networks, poor channel quality may lead to packet loss. In some cases, some packets may be lost suddenly on a wireless link [44]. This kind of loss is called a burst erasure. The burst erasure channels can be modeled using the well-known Gilbert–Elliott model [45], which is based on a Markov chain with two states, “Bad” (B) and “Good” (G). Assume that $h_{b}$ and $h_{g}$ are the probabilities of making no error in state B and G. Especially, $h_{b}=1$ means no packet loss when the channel state is B, and similarly, $h_{g}=1$ means no packet loss when the channel state is G. Therefore, the erasure probabilities in the B and G states can be derived as $1-h_{b}$ and $1-h_{g}$, respectively. The crossover probabilities between the two states are denoted as $p_{bg}$ and $p_{gb}$. Specifically, $p_{bg}$ is the probability of state transition from B to G, and $p_{gb}$ represents the probability of the opposite. It is obvious that the average packet loss rate over this erasure channel can be calculated as in (11):

(11) $$p_{e}=\frac{p_{gb}}{p_{gb}+p_{bg}}\left(1-h_{b}\right)$$

For simplicity of analysis, we use the average packet loss rate as a measure of channel quality ϵ.

**Figure 2:**
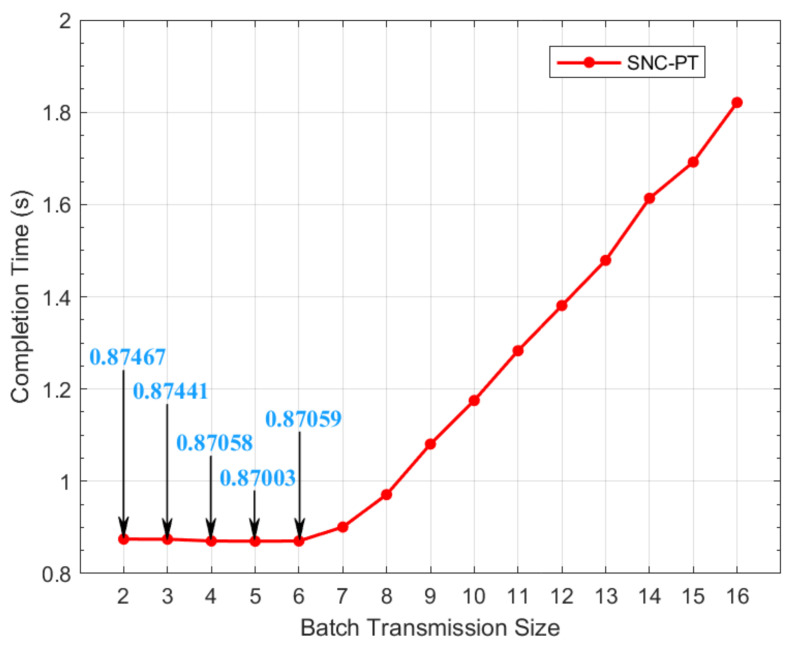
The completion time of SNC-PT with different BTSs.

As mentioned in the previous section, the BTS decides the times of transmission before the next subset is selected in sparse network coding. Eventually, it decides the redundancy of the coding and overhead in the transmission. Hence, we need to find an optimal BTS to be used in SNC-PT that can minimize the overhead and optimize the performance of the SNC. We analyze the completion time of SNC-PT with different BTSs in the study. As shown in Figure 2, the completion time starts increasing linearly when the BTS is larger than 8. The performance in terms of completion time reaches an optimal level, i.e., 0.87003, when the BTS is set to 5. Hence, we set the BTS to 5 in the following study for SNC-PT.

**Figure 3:**
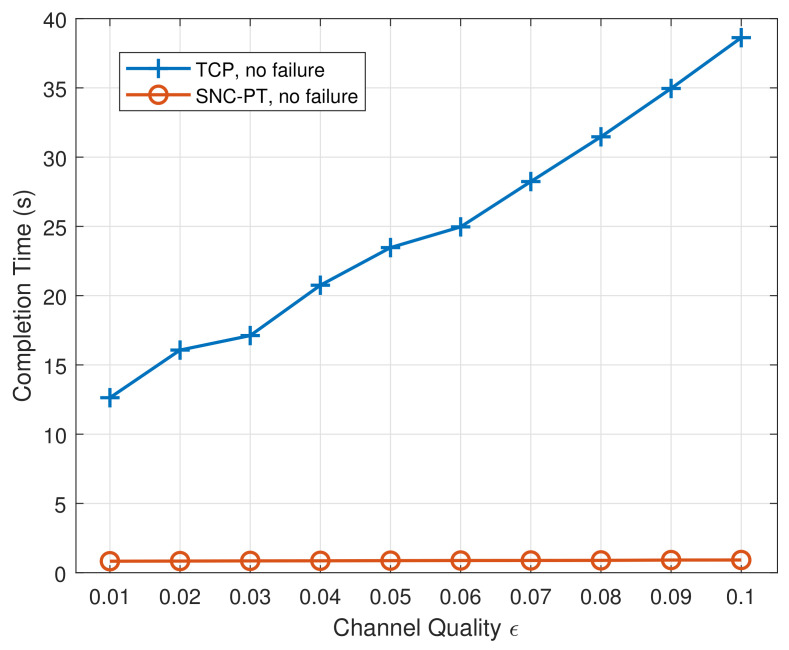
The completion time with different channel qualities in the case of no failure.

The average completion time of the SNC-PT mechanism and TCP transmission is shown in Figure 3. It can be seen that network coding with parallel transmission can significantly reduce the completion time to less than 1s. This is due to the redundancy introduced by the proposed mechanism, which makes it possible for reliable transmission without retransmission. In comparison, TCP tends to mark the loss of packets (i.e., NACK feedback) as congestion and reduces the transmission rate. This rate adjustment usually triggers the additive increase multiple decrease (AIMD) mode. In wireless communication, using the AIMD mode to adjust the rate will reduce the throughput and prolong the transmission completion time. It is because the loss of packets is usually caused by instantaneous fading rather than the congestion of wireless links. Furthermore, the transmission completion time of TCP will be affected in a wireless environment with a long round trip time (RTT) and an unreliable feedback link due to its dependence on the feedback of the receiver to slide the transmission window or on retransmission of lost messages. The SNC-PT mechanism does not need feedback, and there is no congestion or flow control by default. Therefore, its end-to-end transmission time is much less than with TCP. It is worth noting that the loss of encoded packets does not result in the loss of original packets since the redundant encoded packets are employed in the mechanism.

**Figure 4:**
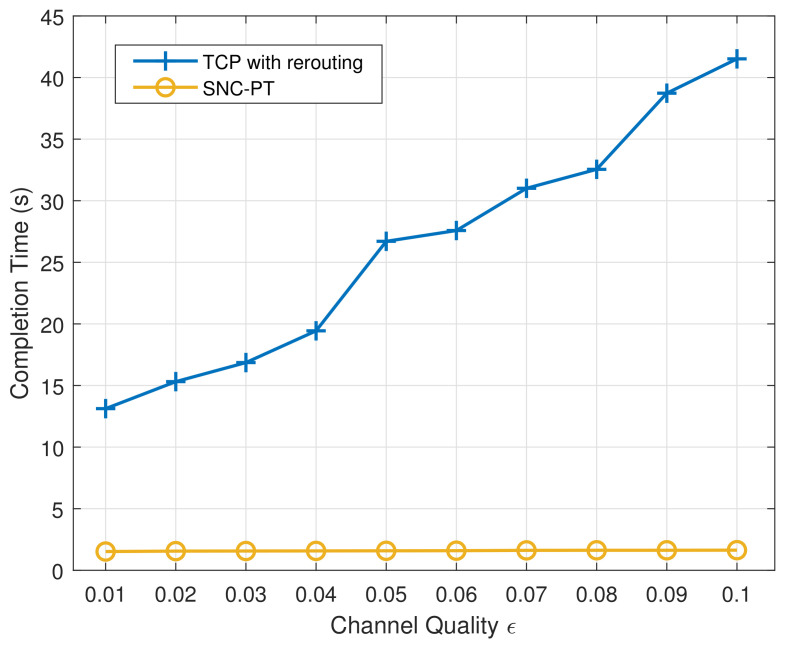
The average completion time with different channel qualities in the case of a single failure.

We further study the performance of SNC-PT in the case of a single failure. As shown in Figure 4, the proposed mechanism still outperforms TCP with rerouting. In particular, it is not affected by the bad channel quality. The completion time remains stable with the SNC-PT mechanism, whereas it increases dramatically with rerouting. Compared with TCP with rerouting, UDP combined with sparse network coding and parallel transmission can achieve a short completion time and reliable transmission in the case of a one path failure and bad channel quality.

**Figure 5:**
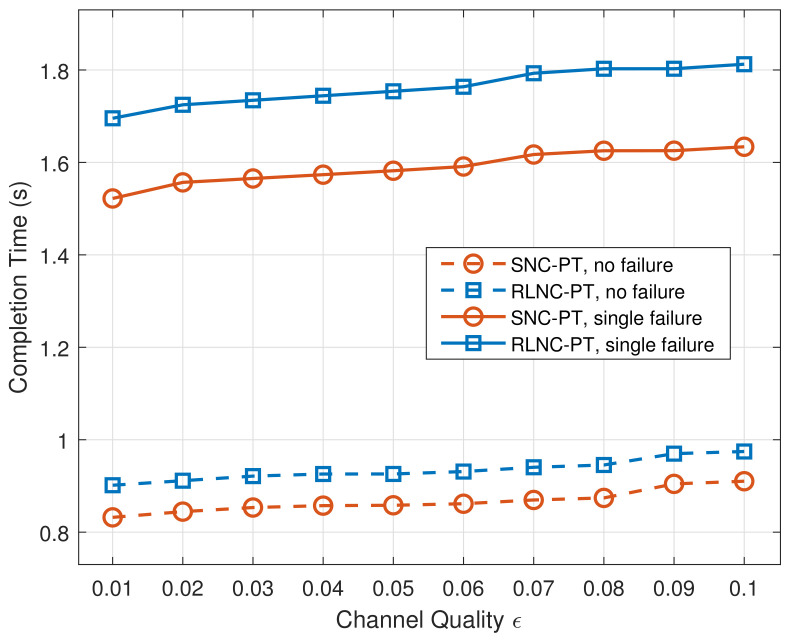
The average completion time of RLNC-PT and SNC-PT in two cases.

The average completion time of the original RLNC and sparse network coding, combined with parallel transmission in two cases with different channel qualities, is presented in Figure 5. Thanks to the parallel transmission, the traffic can still be transmitted through another intact path within an acceptable completion time, even if a node failure in the network causes the interruption on one path. In addition, in two cases, it is obvious that channel quality has little effect on both mechanisms. The performance of SNC-PT is slightly better than that of RLNC-PT.

**Figure 6:**
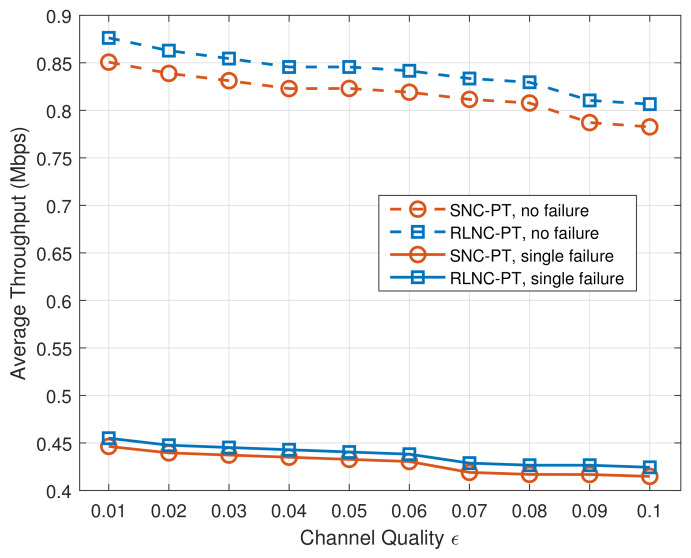
Throughput with different channel qualities in two cases.

The average throughput performance of our mechanisms with different channel qualities in the two cases is shown in Figure 6. When one path fails, the average throughput of both the RLNC-PT and SNC-PT methods decreases by approximately half. This is because the source node equally sends encoded packets through the two paths selected in advance, resulting in the loss of almost half of the encoded packets. Although the average throughput of SNC-PT is slightly poorer than that of RLNC-PT, the goodput of SNC-PT outperforms RLNC-PT, as discussed in the following.

**Figure 7:**
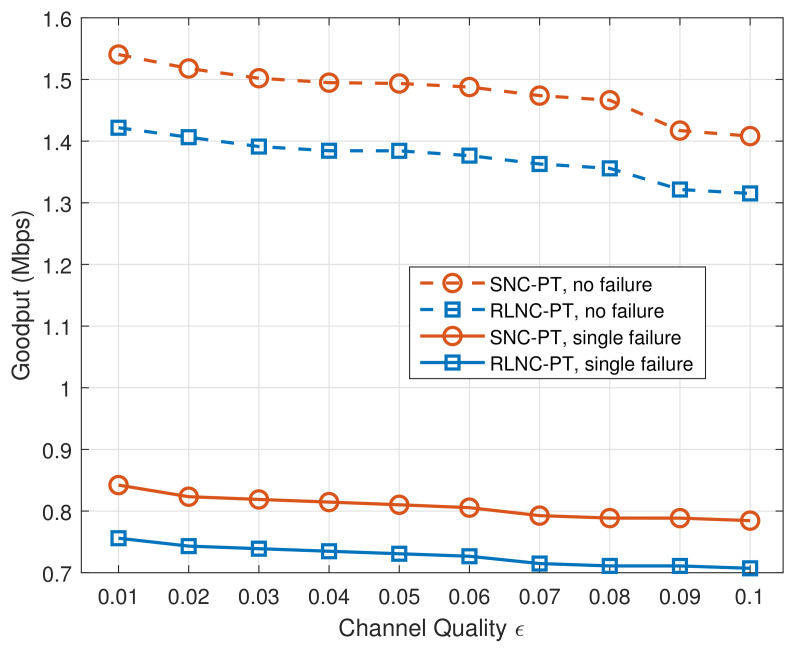
Goodput with different channel qualities in two cases.

We consider the goodput at the application layer level. This QoS performance metric counts the number of useful bits per unit time forwarded from a source address to a destination address in the network within the recovery time, excluding protocol overhead and retransmitted packets. Figure 7 shows the average goodput of our mechanism in two cases. SNC-PT performs better than RLNC-PT, even when the channel quality is poor. With a single failure, the goodput of both mechanisms is worse than the performance in the scenario of no failures. However, both mechanisms can achieve decent goodput. When the channel quality is bad, e.g., ϵ=0.1, SNC-PT is observed with better goodput compared with RLNC-PT. This is due to the lower complexity of the coding as well as less overhead.

**Figure 8:**
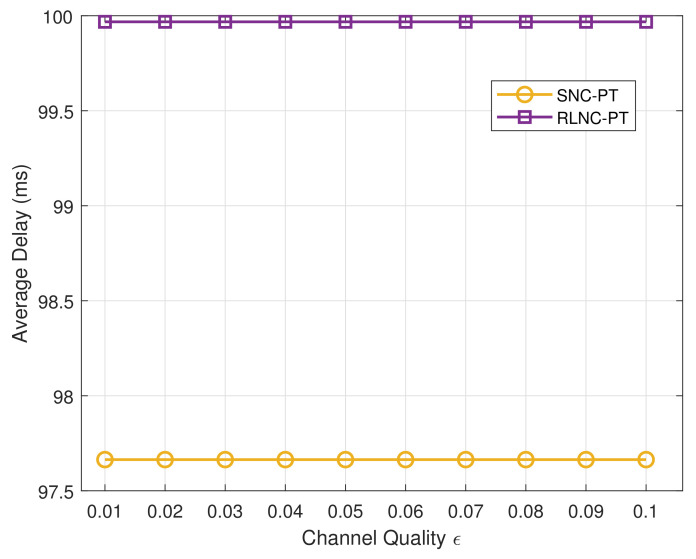
The average delay with different channel qualities in the case of a single failure.

Figure 8 presents the average delay of the two mechanisms. It can be seen that the delay performance of both coding methods is not affected by channel quality in case of a path failure. Although RLNC-PT is observed with a longer delay, the average delays of both mechanisms are under 100 ms.

## 5. Conclusions

In this paper, we proposed a fault-tolerant transmission scheme tailored for an SDN-based IIoT over FiWi networks, which considered the fault tolerance of both the wired and wireless segments. The proposed scheme is based on network coding and parallel transmission. We presented two mechanisms, referred to as RLNC-PT and SNC-PT, and systematically analyzed the performance of the two mechanisms. The numerical results showed that both the RLNC-PT and SNC-PT outperformed TCP with rerouting, which is the commonly adopted solution in the literature. Thanks to the lower coding complexity and overhead, the SNC-PT achieved better performance in terms of goodput, completion time, and average delay. However, both mechanisms demonstrated fault tolerance against node failure and packet loss caused by poor channel quality. Finally, we also showed that the average delay of the proposed mechanisms was very small. The proposed scheme with two fault-tolerant mechanisms is a promising solution for emerging IIoT applications with QoS requirements of high reliability and low latency.

## Data Availability

Not applicable.
